# No association between gluten sensitivity and amyotrophic lateral sclerosis

**DOI:** 10.1007/s00415-017-8400-8

**Published:** 2017-02-06

**Authors:** Anne E. Visser, Raha Pazoki, Sara L. Pulit, Wouter van Rheenen, Joost Raaphorst, Anneke J. van der Kooi, Isis Ricaño-Ponce, Cisca Wijmenga, Henny G. Otten, Jan H. Veldink, Leonard H. van den Berg

**Affiliations:** 1 Department of Neurology, Brain Center Rudolf Magnus, F02.230, University Medical Center Utrecht, P.O. Box 85500, 3508 GA Utrecht, The Netherlands; 2 Department of Epidemiology and Biostatistics, MRC-PHE Center for Environment and Health, School of Public Health, Imperial College London, London, UK; 3 Department of Neurology, Donders Institute for Brain, Cognition and Behavior, Center for Neuroscience, Radboud University Nijmegen Medical Center, Nijmegen, The Netherlands; 4 Department of Neurology, Amsterdam Medical Center, University of Amsterdam, Amsterdam, The Netherlands; 5 Genetics Department, University of Groningen, University Medical Center Groningen, Groningen, The Netherlands; 6 Laboratory of Translational Immunology, University Medical Center Utrecht, Utrecht, The Netherlands

**Keywords:** Amyotrophic lateral sclerosis, Gluten, Epidemiology, Serology, Genome-wide association studies

## Abstract

To examine evidence for a role of gluten sensitivity (GS) or celiac disease (CD) in ALS etiology, we included participants from a population-based case–control study in The Netherlands between January 2006 and December 2015. We compared levels and seroprevalence of IgA antibodies to tissue transglutaminase 6 (TG6) in 359 ALS patients and 359 controls, and to transglutaminase 2 (TG2) and endomysium (EMA) in 199 ALS patients and 199 controls. Questionnaire data on 1829 ALS patients and 3920 controls were examined for CD or gluten-free diets (GFD). Genetic correlation and HLA allele frequencies were analyzed using two genome-wide association studies: one on ALS (12,577 cases, 23,475 controls), and one on CD (4533 cases, 10,750 controls). We found one patient with TG6, TG2 and EMA antibodies who had typical ALS and no symptoms of GS. TG6 antibody concentrations and positivity, CD prevalence and adherence to a GFD were similar in patients and controls (*p* > 0.66) and in these patients disease progression was compatible with typical ALS. CD and ALS were not found to be genetically correlated (*p* > 0.37). CD-associated HLA allele frequencies were similar in patients and controls (*p* > 0.28). In conclusion, we found no serological evidence for involvement of gluten-related antibodies in ALS etiology nor did we observe an association between CD and ALS in medical history or genetic data, indicating that there is no evidence in our data for an association between the two diseases. Hence, a role for a GFD in the ALS treatment seems unlikely.

**Electronic supplementary material:**

The online version of this article (doi:10.1007/s00415-017-8400-8) contains supplementary material, which is available to authorized users.

## Introduction

Recent studies have proposed a potential link between celiac disease (CD) and ALS [1, 2], and suggest that sensitivity to gluten (GS) may occur in a subgroup of patients, who may then benefit from a gluten-free diet (GFD) [2]. The efficacy of GFD in seropositive patients is currently being investigated [3].

These epidemiological observations suggest that CD and ALS may share (genetic) susceptibility factors. CD has a clear genetic component (heritability ~80% [4]), and genome-wide association studies (GWAS) have revealed >40 common-variant loci associated with CD; the strongest associated with the human leukocyte antigen (HLA) genes, which lie in the major histocompatibility complex (MCH) [5]. In contrast, in ALS, which has a heritability of ~65% [6], GWAS revealed a total of 7 loci [7]. Despite these distinct architectures, overlap between immune-regulated and neurological disease is not unheard of: CD can present with neurological symptoms [8–13], and frontotemporal dementia, which has several risk loci that overlap with those of ALS [14], shows an additional risk variant in the MHC [15].

If CD and ALS are causally related, one can expect a significant overlap in genetic architectures; methods to estimate this overlap are currently available [16, 17]. The association between GS or CD and ALS may have implications for our understanding of a pathogenic mechanism, as well as for future ALS treatment options. In this study, therefore, we combined population-based case–control data on gluten-related antibodies with medical history and dietary information, and large-scale genetic data from GWAS on CD and ALS, to examine the evidence for the role of GS or CD in ALS etiology.

## Methods

### Study population

All participants in the serological and questionnaire studies were included in the Prospective ALS study the Netherlands (PAN) between 1 January 2006 and 31 December 2015 [18]. Patients were diagnosed according to the revised El Escorial criteria [19]. The population-based design of the PAN study was ensured by inclusion of patients through multiple sources: neurologists, rehabilitation physicians, the Dutch neuromuscular Patient Association and our ALS website. Age- (±5 years) and gender-matched controls were included through the general practitioner of the participating patient. In the Netherlands, every resident is registered at a general practitioner, which makes the sample representative of the general population.

For genetic correlation analyses, we used data from a GWAS of ALS, which included 12,577 sporadic ALS cases and 23,475 unaffected controls, and a GWAS of CD, which included 4533 patients and 10,750 controls. Both studies comprised cohorts from Europe and the USA [7, 20].

Separately, ALS cases and population-matched controls are being collected worldwide as part of Project MinE [21]. A subset of these samples is drawn from the neuromuscular clinic at the University Medical Center Utrecht and controls are selected from the PAN study to match cases by geography, age and gender. Data collected on the samples include disease status and genotyping on the Illumina 2.5M array. As part of the Project MinE, samples and variants have undergone quality control (detailed description in Online Resource 1) [7].

### Serological testing

In 359 population-based patients and 359 matched controls, we measured TG6 antibody concentrations using the ELISA IgA kit (Zedira, Darmstadt, Germany). TG6 positivity was calculated using the mean of the control group (17.33 U/mL) plus 2 standard deviations (2 × 19.59 U/mL), and was set at 56.51 U/mL. In a subset of 199 patients and 199 controls, antibodies to TG2 were measured using the ELiA Celikey IgA kit (Phadia AB, Uppsala, Sweden). Serum samples containing a TG2 antibody titer of more than 10 U/mL were considered positive, as recommended by the manufacturer. In the same subset, we determined the prevalence of IgA class endomysial antibodies (EMA) using indirect immunofluorescence (Biognost, Heule, Belgium). Total IgA was measured, and a serum IgA concentration below 0.05 g/L was regarded as IgA deficiency. Gluten-associated antibodies were compared between groups using Student’s *t* test for antibody concentrations and the Chi-square test for comparison of the seroprevalence, or Fisher’s exact test in case of cell counts below five. Due to low numbers, we were unable to test for interaction with gender using an interaction term in a logistic model; instead, we stratified according to gender. We compared antibody levels and positivity between bulbar and spinal patients, and antibody measures in patients with a time to diagnosis within the first quartile (representing patients with a rapid disease course) with controls. Clinical characteristics between groups were compared using the Mann–Whitney *U* test for continuous characteristics; the Chi square for categorical characteristics (or Fisher’s exact test as appropriate); the Cox proportional hazard model for survival.

### Medical history and gluten-free diet

We examined a structured questionnaire collected from 1829 population-based ALS patients and 3920 matched controls to collect medical histories [18]. For patients, we analyzed data referring to the period before symptom onset. In addition, in the 199-item food frequency questionnaire, participants were asked about their adherence to a specific diet [22]. For patients, this was defined as the 1-month period before symptom onset. All questionnaires were checked for missing data or inconsistencies, and participants were contacted by telephone to complete or correct the data. The survival status of patients was obtained by checking the municipal population register and/or contacting the general practitioner every 3 months. Proportions of participants with CD or a GFD were compared using the Fisher’s exact test because of the small numbers.

### Genetic correlation using LD score regression

Correlations in genetic architectures between two traits can provide insight into potential overlapping etiology between diseases and may help disentangle shared potential causal mechanisms [17]. To estimate the genome-wide genetic correlation between ALS and CD, we used Linkage Disequilibrium Score Regression (LDSC). In short, LDSC considers the fact that, for polygenic traits, the association signal at a single-nucleotide polymorphism (SNP) will also capture information for SNPs nearby in linkage disequilibrium. This relationship between linkage disequilibrium and association signal can also be used to test for relationships between two traits at all SNPs across the genome. A GWAS of ALS had been performed using a linear mixed model (LMM) implemented in the Genome-Wide Complex Trait Analysis (GCTA) software [7, 23], from which the summary statistics for 1,217,311 SNPs were available [7]. Summary statistics for 499,513 SNPs from the CD GWAS were obtained via personal communication with the authors. Details of the statistical LDSC analysis are provided in the Online Resource 2.

### Genetic correlation using genetic risk scores

We constructed a genetic risk score from associated CD SNPs, to see if such a score could help predict ALS disease status. We downloaded all SNPs associated by GWAS to CD at *p* < 1 × 10^−6^ from the NHGRI GWAS Database [24]; data downloaded included SNP rsID, chromosome, position, risk allele, *P* value, and reported odds ratio from the GWAS. A total of 31 SNPs had complete information. For duplicate SNPs, we kept the SNP results from the study with the largest sample size. We extracted the genotypes for these SNPs from 2833 unrelated Dutch samples (1823 cases and 1010 controls) that had been genotyped on the Illumina 2.5 M array as part of Project MinE. A total of 21 SNPs were found in the genotyping data. We LD-pruned the SNPs (*r* ^2^ < 0.05) to ensure that all SNPs represented independent associations to CD; LD pruning removed two SNPs. We used the genotypes for the remaining 19 SNPs to construct a genetic risk score by weighting the genotypes by the odds ratio reported in the CD GWAS and summing across all weighted genotypes (performed in Plink). We used logistic regression to test for association between ALS disease status and CD-based genetic risk score, correcting for ten principal components and gender.

### HLA association testing

The same 2833 unrelated Dutch samples from Project MinE were used to perform imputation of the MHC. MHC genotypes (build hg19, chromosome 6, positions 24,092,021–38,892,022) for these samples were extracted from genotypes generated using the Illumina 2.5 M genotyping array. Details on sample quality checks and imputation of the MHC genotypes are provided in the Online Resource 3. When imputation was complete, we performed QC, removing any sample with >2.5 alleles at any HLA allele (Online Resource 3). We used the resulting genotype dosages in 1786 ALS cases and 999 controls to perform association testing between ALS status and four CD-associated HLA alleles: HLA-DQA1*0501, HLA-DQB1*0201, HLA-DQB1*0202, and HLA-DQB1*0302. We adjusted for ten principal components and gender. The fifth HLA allele of interest, HLA-DQA1*0505, could not be tested as it was absent from the SNP2HLA imputation reference panel.

## Results

### Serological testing

We tested TG6 antibody levels in 359 patients and 359 controls, matched for baseline characteristics (Table 1). The subgroup of 199 patients and 199 controls with measured TG2 antibody and EMA levels were also characteristic-matched (data not shown). None of the patients was IgA deficient. TG2 positivity (>10 U/mL) was found in one patient and zero controls. This patient was also the only participant who tested positive for EMA, and was one of the eleven patients with positive antibodies to TG6. Despite having TG2, EMA and TG6 antibodies, this patient had clinical characteristics (probable ALS, bulbar onset at the age of 66 years) and a survival (1.33 years from onset) compatible with typical ALS. Furthermore, routine blood tests were normal, indicated no anemia, and magnetic resonance imaging (MRI) revealed no white matter lesions.

**Table 1:** Baseline characteristics

	Serological cohort		Population-based cohort	
	Patients (*n* = 359)	Controls (*n* = 359)	Patients (*n* = 1829)^b^	Controls (*n* = 3920)
Male, *n* (%)	231 (64.3)	217 (60.4)	1096 (59.9)	2334 (59.5)
Age (year), median (range)^a^	64.6 (23.5–87.8)	63.9 (20.4–83.9)	62.8 (23.1–87.9)	63.8 (17.5–91.9)
Age at diagnosis (year), median (range)	65.5 (23.8–88.7)		64.2 (23.8–88.9)	
Bulbar onset, *N* (%)	122 (34.0)		574 (32.2)	
El Escorial criteria, *N* (%)				
Definite	72 (20.1)		323 (18.4)	
Probable	127 (35.4)		724 (41.3)	
Laboratory-supported probable	105 (29.2)		324 (18.5)	
Possible	55 (15.3)		383 (21.8)	

^a^Age at onset for patients; age at inclusion for controls

^b^Missing data in patients: age 54 (3.0%); age at diagnosis 17 (0.9%); site of onset 49 (2.7%); El Escorial criteria 75 (4.1%) of patients. No missing data in controls or in the serological cohort

Patients and controls had comparable TG6 antibody concentrations (Student’s *t* test *p* = 0.66, Table 2) and positivity for TG6 antibodies [11 patients (3.1%) and 12 controls (3.3%), Chi-square test *p* > 0.99]. Stratifying by gender did not reveal a difference in antibody concentrations or prevalence (Student’s/Fisher’s test all *p* > 0.48, data not shown). Clinical characteristics of the 11 seropositive patients were similar to those of the 348 seronegative patients (Table 3). Eighty-one patients with a short time to diagnosis (defined as <6 months and used as a surrogate variable for a rapid disease course) had TG6 antibody levels similar to controls [mean (SD): 21.16 (31.56) vs. 17.33 (19.59), Student’s *p* = 0.30]. Two of the 81 patients (2.5%) tested positive for TG6 antibodies (Fisher’s *p* > 0.99; data not shown). Similarly, bulbar and spinal patients had comparable TG6 antibody levels [mean (SD): 17.53 (27.37) vs. 18.30 (20.20), Student’s *p* = 0.79] and positivity [*n* (%): 3 (2.5%) vs. 8 (3.4%), Fisher’s *p* = 0.76; data not shown].

**Table 2:** Serological results

	Patients (*n* = 359)	Controls (*n* = 359)	*p* value
TG6 IgA, U/mL, mean (SD)	18.1 (22.9)	17.3 (19.6)	0.66
Positive TG6 IgA, *n* (%)^a^	11 (3.1)	12 (3.3)	>0.99

^a^Cutoff is 56.51 U/mL

**Table 3:** Patient characteristics of seropositive and seronegative TG6 patients

	Seropositive (*n* = 11)	Seronegative (*n* = 348)	*p* value
Male, *n* (%)	8 (72.7)	223 (64.1)	0.79
Age (year), median (range)	63.1 (45.1–73.5)	64.8 (23.5–87.8)	0.37
Age at diagnosis (year), median (range)	64.0 (47.1–82.5)	65.6 (23.8–88.7)	0.53
Bulbar onset, *n* (%)	3 (27.3)	119 (34.2)	0.76
Survival (year), median (range)	2.32 (1.17–6.10)	2.32 (0.41–6.76)	0.63
El Escorial criteria, *n* (%)			0.54
Definite	3 (27.3)	69 (19.8)	
Probable	5 (45.5)	122 (35.1)	
Laboratory-supported probable	1 (9.1)	104 (29.9)	
Possible	2 (18.2)	53 (15.2)	

### Medical history and gluten-free diet

Questionnaire-derived data of 1829 ALS patients and 3920 controls showed they had similar characteristics (Table 1). According to the data, 3 of 1829 ALS patients (0.16%) and 4 of 3920 controls (0.10%) had been diagnosed with CD (Fisher’s *p* = 0.69). 1470 of these 1829 patients and 3683 of the 3920 controls filled in a food frequency questionnaire; in addition to the 3 patients and 4 controls with a medical history of CD, we identified 2 more patients (*n* = 5 in total; 0.3%) and 8 controls (*n* = 12 in total; 0.3%) who were following a GFD prior to disease onset (Fisher’s *p* > 0.99). Table 4 shows the clinical characteristics and disease course of the 5 patients who adhered to a GFD. On average, their survival after disease onset was 3.3 years, consistent with standard ALS disease progression.

**Table 4:** Patient characteristics of the five patients on a gluten-free diet

Patient	Gender	Site of onset	Age at onset (year)	Age at diagnosis (year)	EEC	Survival^a^ (year)
1.	Female	Spinal	64.3	64.9	LSP	4.1
2.	Female	Spinal	55.2	56.1	Probable	3.3
3.	Male	Spinal	61.2	63.2	Definite	4.1
4.	Male	Spinal	49.4	49.8	LSP	3.5
5.	Female	Bulbar	64.7	65.1	Probable	1.6

*EEC* El Escorial criteria, *LSP* laboratory-supported probable

^a^All patients were deceased

### Genetic correlation between ALS and CD

We applied LDSC analysis using summary-level results from a GWAS of ALS (12,577 cases and 23,475 controls) and of CD (4533 CD cases and 10,750 controls). The genetic correlation between ALS and CD was 0.06 (standard error = 0.07; *p* = 0.37), indicating that, genome-wide, there was no evidence for genetic correlation between the traits. While LDSC looks for trait correlation, genetic risk scores can be used to test for association between two traits at specific loci. We tested a genetic risk score based on CD loci in the Dutch Project MinE samples, including 1823 ALS cases and 1010 matched controls. The score was not associated with ALS disease status (*p* = 0.47).

### Association testing at HLA alleles

We performed HLA imputation and association analysis in the same Project MinE Dutch ALS patients and controls. After additional QC of the data, we found no association at any of the four HLA alleles associated with CD in 1786 ALS cases and 999 controls (all *p* > 0.28, Table 5).

**Table 5:** HLA allele frequencies

	Dutch ALS patients (*n* = 1786)	Controls (*n* = 999)	*p* value
HLA-DQA1*0501 (%)	25.9	27.1	0.28
HLA-DQB1*0201 (%)	14.4	15.6	0.34
HLA-DQB1*0202 (%)	7.2	6.6	0.61
HLA-DQB1*0302 (%)	10.0	9.4	0.44

## Discussion

In this study, we combined serological, epidemiological and genetic data to examine whether GS or CD is associated with ALS risk. Although we cannot rule out CD in our patient with gluten-related antibodies without performing a small intestine biopsy (gold standard [9]), CD is highly unlikely since blood tests did not indicate anemia [8, 9], brain MRI did not show white matter changes [10–12], and the patient had typical ALS symptoms and disease course. Moreover, whereas our patient experienced speech difficulties, previous case reports do not describe bulbar symptoms in gluten-related ALS mimic syndromes [10–13]. Furthermore, combining two large GWAS revealed no genetic correlation between CD and ALS, either genome-wide or through a targeted approach. HLA alleles in the MHC shown to be associated with CD showed no association with ALS disease status. In addition, we found no difference in concentrations or positivity of gluten-related TG6 antibody between patients and controls. Patients with antibodies to TG6 or CD/GFD have characteristics compatible with ALS and patients on a GFD did not demonstrate prolonged survival time (average survival 3.3 years). In short, across this myriad of analyses, we found no evidence for association or overlap between GS or CD and ALS, and no evidence that initiation of a GFD in ALS patients would be beneficial.

TG6 has been relatively recently added to the list of gluten-related antibodies and, since then, has been described in patients with idiopathic cerebellar ataxia, myelopathy secondary to malabsorption, autoimmune neuropathy and ALS [2, 25, 26]. Using the same ELISA kit for TG6 antibodies as in this study, a previous study in Israeli-descent samples showed 15.3% of the 150 referred patients and 4.3% of the 115 controls to have elevated levels of antibodies to TG6 [2]. In a larger study, we found that 3.1% of 359 patients and 3.3% of 359 controls had elevated levels of antibodies to TG6. The TG6 antibody concentrations of the Israeli controls (21.0 U/mL) and the Dutch controls (17.3 U/mL) were similar, but cutoff scores for positivity differed: 42 U/mL in the study by Gadoth et al. [2] and 56.51 U/mL in our study. This could explain the slightly lower frequency of controls with elevated TG6 antibodies in our study. On the other hand, the TG6 antibody concentrations in patients in the Israel-based study [2] (29.3 U/mL) seem to be higher compared to those in this study (18.1 U/mL). One similarity between the two studies is the prevalence of the CD-associated HLA risk alleles, which were not different between patients and controls. Thus, although the prevalence of CD in Israel does not seem to be higher than in Europe [27], it might be that GS, as measured by the presence of TG6 antibodies, occurs in a subgroup of Israeli ALS patients and not in patients of Dutch/European origin.

The CD prevalence of 0.10–0.16%, as reported by questionnaires in our study, is lower than that of the European population (around 1%) [8]. We expect, however, this bias to be non-differential between patients and controls, i.e., not related to the presence of disease. In addition, we did not see a difference between patients and controls who are on a GFD as reported in the food frequency questionnaire, a finding in line with a nationwide record-linkage study by Ludvigsson et al., in which the number of ALS diagnoses during follow-up among individuals diagnosed with CD via small intestinal biopsy was similar to the number of ALS diagnoses in the general Swedish population [28]. Using the same study design as Ludvigsson et al., Turner et al. found higher than expected numbers of ALS patients among groups of individuals with various autoimmune diseases, one of which was CD [1]. This finding contradicts the study by Seelen et al. in which no prevalence difference of autoimmune diseases was found between cases and controls [29].

CD is triggered upon exposure to gluten. In this study, we chose to investigate IgA class antibodies specifically, as gluten-related serology tests for TG2 and EMA are usually IgA based and the previous study on TG6 antibodies in relation to ALS showed IgA, as opposed to IgG class antibodies, to be relevant [2, 9]. Based on positive associations published in case reports, a previous study suggested including serological tests for GS or CD in the diagnostic workup for ALS [10]. The results of our study reveal that there is no indication for initiating a GFD in patients who have positive antibody tests for TG6, TG2 or EMA and typical ALS without features of CD, such as anemia or MRI white matter changes. The diet can be burdensome and limit quality of life [30, 31], a heavy price to pay for a patient with a fatal disease. Moreover, a phase 2 trial demonstrated the benefits of a high-calorie diet in ALS patients [32], something that might be difficult to achieve with a GFD.

In the present relatively large case–control study, we found no serological evidence for involvement of gluten-related antibodies in ALS etiology. We did not observe an association between CD and ALS in either medical history or genetic data analysis. These findings indicate that, in our data, there is no evidence for a causal relationship between GS or CD and ALS, and, therefore, it is unlikely that there is a role for a GFD in the treatment of ALS.

## Electronic supplementary material

Below is the link to the electronic supplementary material.

Supplementary material 1 (PDF 61 kb)

Supplementary material 2 (PDF 59 kb)

Supplementary material 3 (PDF 66 kb)
