# Supplementary material for: No association between gluten sensitivity and amyotrophic lateral sclerosis
Source: J Neurol. 2017 Feb 6;264(4):694–700. doi: 10.1007/s00415-017-8400-8 (PMC5374172; doi:10.1007/s00415-017-8400-8)
Supplement: Supplementary file 1 — Supplementary material 1 (PDF 61 kb) [file 415_2017_8400_MOESM1_ESM.pdf]

Online Resource 1

Quality control of the Project MinE samples and variants
Samples included in Project MinE underwent whole-genome sequencing, genotyping on the Illumina 2.5M, and methylation data collection. Samples underwent QC based on the whole-genome sequencing data.

Samples were checked for the following metrics (with exclusion criteria in parentheses): total depth (no exclusion necessary), transition/transversion ratio (>2.09), concordance with genotype data (<95%), phenotype/genotype sex concordance (discordant samples are removed), overall missingness (>5%), inbreeding (>3 standard deviations from the distribution), relatedness (duplicates are removed; related samples with kinship > 0.0625 are dropped for analyses requiring independent samples), total single nucleotide variants (> 4M), total indels (>900k), total singletons (>6 standard deviations from cohort distribution), and ratio of heterozygotes to homozygous non-reference genotypes (>3).

Variants are checked for the following metrics (with exclusion criteria in parentheses): depth of coverage (<1.5x/sample or >34.8x/sample), differential missingness ($p < 10^{-3}$), Hardy-Weinberg equilibrium violations ($p < 10^{-3}$), missingness (>5%), and overall “PASSing rate” (according to Illumina genotype calling pipeline which is performed on a sample-level basis, <70% considering all samples).
