# Supplementary material for: No association between gluten sensitivity and amyotrophic lateral sclerosis
Source: J Neurol. 2017 Feb 6;264(4):694–700. doi: 10.1007/s00415-017-8400-8 (PMC5374172; doi:10.1007/s00415-017-8400-8)
Supplement: Supplementary file 2 — Supplementary material 2 (PDF 59 kb) [file 415_2017_8400_MOESM2_ESM.pdf]

Online Resource 2

Detailed description of the LD Score Regression (LDSC) analysis to estimate genetic correlation between ALS and CD

Genetic correlation between ALS and CD was calculated using LDSC regression software v1.0.0 which measures genetic correlation between traits accounting for confounding biases, such as linkage disequilibrium, cryptic relatedness and population stratification.[1] We used pre-calculated LD scores and regression-weighted LD scores for 1,293,150 SNPs using the European samples of the 1000 Genome project (https://data.broadinstitute.org/alkesgroup/LDSCORE/eur_w_ld_chr.tar.bz2 and https://data.broadinstitute.org/alkesgroup/LDSCORE/w_hm3.snplist.bz2 access date: 9 November 2016). After merging the summary statistics from the ALS and CD GWAS, summary-level statistics were available from both traits for 488,242 valid alleles and included in the LDSC analysis.

We constrained the heritability intercept of ALS to 1 because the ALS GWAS was performed using an LMM and with this method, population stratification and inflation of test statistics is minimal. Since CD was analyzed using a regular logistic regression, inflation of test statistics due to population substructures could not be rejected. Therefore, we left the heritability intercept for CD free.

Reference

1. Bulik-Sullivan BK, Loh PR, Finucane HK, et al (2015) LD score regression distinguishes confounding from polygenicity in Genome-Wide Association Studies. Nat Genet 47(3):291-295.
