# Supplementary material for: No association between gluten sensitivity and amyotrophic lateral sclerosis
Source: J Neurol. 2017 Feb 6;264(4):694–700. doi: 10.1007/s00415-017-8400-8 (PMC5374172; doi:10.1007/s00415-017-8400-8)
Supplement: Supplementary file 3 — Supplementary material 3 (PDF 66 kb) [file 415_2017_8400_MOESM3_ESM.pdf]

Online Resource 3

Imputation of and association testing in the MHC
We performed imputation of the MHC in Dutch-ancestry samples genotyped on the Illumina 2.5M array as part of Project MinE. Only samples that passed quality control of the whole-genome sequencing data were considered for analysis. Before performing imputation, we carried out quality control on the genotyping data. We selected out all SNPs within the MHC (defined as position 24,092,021 to position 38,892,022 on chromosome 6, build hg19).

Samples with > 10% missingness across the MHC were dropped. SNPs out of Hardy-Weinberg equilibrium ($p < 1 \times 10^{-6}$), with significant differential missingness between cases and controls ($p < 1 \times 10^{-6}$), overall missingness > 1%, or with frequency < 1% were removed. This left 3,614 SNPs for imputation. We then imputed the MHC using the SNP2HLA pipeline.[1] In short, the pipeline uses a reference panel generated by the Type I Diabetes Genetics Consortium (T1DGC) and is comprised of SNPs, amino acids, and HLA types collected in 5,225 samples. SNP2HLA uses BEAGLE and the T1DGC reference panel to impute amino acids and HLA types into samples genotyped on commercial arrays.

When the imputation is complete, it results in a 'dosage' for every sample at every SNP, amino acid and HLA allele. The dosage is a continuous number between 0 and 2. At each HLA allele, we tested the total sum of alleles carried by an individual at that allele. Samples with > 2.5 alleles (introduced by imprecision in the imputation) were removed from the analysis; a total of 48 samples were failed, leaving 1,786 ALS cases and 999 controls. We checked the imputation quality (r^2) of all of the CD-associated alleles being tested. All alleles had an $r^2 > 0.9$, indicating high-quality imputation at these alleles. We performed association testing at these alleles using a logistic regression implemented in Plink, adjusting for the top ten principal components and gender.

Reference

1. Jia X, Han B, Onengut-Gumuscu S, et al (2013) Imputing amino acid polymorphisms in human leukocyte antigens. PLoS One 8(6):e64683.
